# Mechanism of a rabbit monoclonal antibody broadly neutralizing SARS-CoV-2 variants

**DOI:** 10.1038/s42003-023-04759-5

**Published:** 2023-04-03

**Authors:** Hangtian Guo, Yixuan Yang, Tiantian Zhao, Yuchi Lu, Yan Gao, Tinghan Li, Hang Xiao, Xiaoyu Chu, Le Zheng, Wanting Li, Hao Cheng, Haibin Huang, Yang Liu, Yang Lou, Henry C. Nguyen, Chao Wu, Yuxin Chen, Haitao Yang, Xiaoyun Ji

**Affiliations:** 1 The State Key Laboratory of Pharmaceutical Biotechnology, School of Life Sciences, Nanjing University, Nanjing, Jiangsu 210023 China; 2 Department of Infectious Diseases, Nanjing Drum Tower Hospital Clinical College of Nanjing University of Chinese Medicine, Nanjing, Jiangsu 210008 China; 3 Shanghai Institute for Advanced Immunochemical Studies and School of Life Science and Technology, ShanghaiTech University, Shanghai, 201210 China; 4 Shanghai Clinical Research and Trial Center, Shanghai, 201210 China; 5 Yurogen Biosystem LLC, Wuhan, Hubei 430075 China; 6 Department of Infectious Diseases, Nanjing Drum Tower Hospital Clinical College of Xuzhou Medical University, Nanjing, Jiangsu 210008 China; 7 Institute of Viruses and Infectious Diseases, Nanjing University, Nanjing, Jiangsu 210008 China; 8 Department of Laboratory Medicine, Nanjing Drum Tower Hospital, Nanjing University Medical School, Nanjing, Jiangsu 210008 China

**Keywords:** Cryoelectron microscopy, Proteins

## Abstract

Due to the continuous evolution of SARS-CoV-2, the Omicron variant has emerged and exhibits severe immune evasion. The high number of mutations at key antigenic sites on the spike protein has made a large number of existing antibodies and vaccines ineffective against this variant. Therefore, it is urgent to develop efficient broad-spectrum neutralizing therapeutic drugs. Here we characterize a rabbit monoclonal antibody (RmAb) 1H1 with broad-spectrum neutralizing potency against Omicron sublineages including BA.1, BA.1.1, BA.2, BA.2.12.1, BA.2.75, BA.3 and BA.4/5. Cryo-electron microscopy (cryo-EM) structure determination of the BA.1 spike-1H1 Fab complexes shows that 1H1 targets a highly conserved region of RBD and avoids most of the circulating Omicron mutations, explaining its broad-spectrum neutralization potency. Our findings indicate 1H1 as a promising RmAb model for designing broad-spectrum neutralizing antibodies and shed light on the development of therapeutic agents as well as effective vaccines against newly emerging variants in the future.

## Abstract (web summary)

The molecular mechanism of broad-spectrum neutralization of a rabbit monoclonal antibody 1H1 against Omicron sublineages is revealed by cryo-EM.

## Introduction

After being reported in Southern Africa in late November 2021, the variant of severe acute respiratory syndrome coronavirus 2 (SARS-CoV-2) named Omicron (sublineage BA.1) has spread worldwide and raised serious concerns due to the unprecedented number of mutations it harbors in the spike protein^1–4^. As the most evolutionarily distinct variant of concern (VOC), Omicron displays higher transmissibility and enhanced immune evasion compared to other SARS-CoV-2 variants^5–10^. In addition, the continuous evolution of SARS-CoV-2 has given rise to the emergence of Omicron sublineages, including BA.2, BA.2.12.1, BA.2.75, BA.4 and BA.5^11–15^. Particularly, the BA.5 lineage has caused surges in several countries since being first reported in April, 2022^16,17^. To manage Omicron sublineages and prepare for potential emergencies of SARS-CoV-2 VOCs in the future, it is urgent to develop new therapeutic antibodies against all SARS-CoV-2 VOCs. In addition, it is essential to characterize their mechanism for broad-spectrum neutralization.

As the major determinant of the host specificity, the SARS-CoV-2 spike glycoprotein contains S1 and S2 subunits responsible for receptor recognition and membrane fusion, similar to other coronaviruses such as SARS-CoV and the Middle East Respiratory Syndrome Coronavirus (MERS-CoV)^18,19^. Therefore, the spike protein has been a key target for neutralizing monoclonal antibodies (mAbs), especially the receptor-binding domain (RBD) within the S1 subunit^20,21^. The RBD can adopt two different conformations: the “up” conformation, which is receptor-accessible, and the “down” conformation, which is shielded from receptor binding^21^. Currently, there are more than 20 mAbs in clinical trials, and some have been approved by the US Food and Drug Administration (FDA) for the treatment of COVID-19, including Sotrovimab, the combination of Casirivimab and Imdevimab and the combination of Bamlanivimab and Etesevimab^22–25^. The majority of these antibodies target the SARS-CoV-2 RBD and inhibit viral entry by binding to the ACE2 receptor binding motif (RBM), directly impeding its binding to the ACE2 receptor^7^. Other antibodies bind outside of the RBM but sterically inhibit ACE2 binding^26^. Based on RBD epitopes, the reported mAbs can be categorized into 4 classes^27,28^. Class 1 mAbs bind up RBDs at the RBM region and overlap with the ACE2 epitope. Class 2 mAbs bind to the RBM region with RBDs in both up and down conformations. Class 3 mAbs bind to both up and down RBDs with epitopes outside the RBM region. Class 4 mAbs can only bind to the up RBD and recognize non-RBM epitopes^22^. In addition, some antibodies may destabilize the spike trimer to neutralize the virus in vivo^29,30^.

One of the current limitations in the development of therapeutic mAbs is the continuous emergence of SARS-CoV-2 VOCs, which carry mutations in their spike proteins that can render a large number of mAbs partially or entirely ineffective^7,9^. Some mutation sites, such as L452 in Omicron sublineages BA.2.12.1 and BA.4/5, and F486 in BA.4/5 exhibit stronger immune evasion capability than those in BA.2^2^. As a result, many authorized or approved therapeutic mAbs have limited applications and are constantly challenged by new variants. For example, S309 (parent of Sotrovimab) and the COV2-2196/COV2-2130 cocktail (parents of Cilgavimab/Tixagevimab) were reported to have reduced potency against Omicron according to pseudovirus or authentic virus assays^6,7,9,31,32^. It has also been reported that although LY-CoV1404 (parent of Bebtelovimab) retained potency against ancestral Omicron variants among clinical mAbs^3,33,34^, it is not currently authorized for emergency use in any U.S. region due to its limited efficacy against Omicron subvariants BQ.1 and BQ.1.1 (https://www.fda.gov/drugs/drug-safety-and-availability).

Previously, we reported four rabbit mAbs (RmAbs) that can effectively neutralize SARS-CoV-2 from immunized rabbits receiving a DNA prime-protein boost immunization strategy^35^. These include 1H1, 5E1, 7G5 and 9H1, with 1H1 exhibiting broad neutralizing activity against six SARS-CoV-2 variants, including D614G, Alpha (B.1.1.7), Beta (B.1.351), Gamma (P.1), Epsilon (B.1.429) and Iota (B.1.526)^35^. Here we further demonstrate the potent and broad neutralization potential of 1H1 with cross-neutralizing potency against Delta (B.1.617.2) and Omicron sublineages, including BA.1, BA.1.1, BA.2, BA.2.12.1, BA.2.75, BA.3 and BA.4/5. We also present cryo-electron microscopy (cryo-EM) structures of the Omicron BA.1 spike ectodomain (ECD) complexed with 1H1 Fabs to demonstrate a unique neutralization mechanism. Furthermore, we find that 1H1 can selectively target SARS-CoV-2 variants while remaining inactive against SARS-CoV and MERS-CoV. Our studies provide insights into the development of antibody-based treatments and rational vaccine design against the widely spread Omicron and newly emerging variants in the future.

## Results

### Identification of the RmAb 1H1 with broad neutralizing potency against Omicron sublineages

**Fig. 1:**
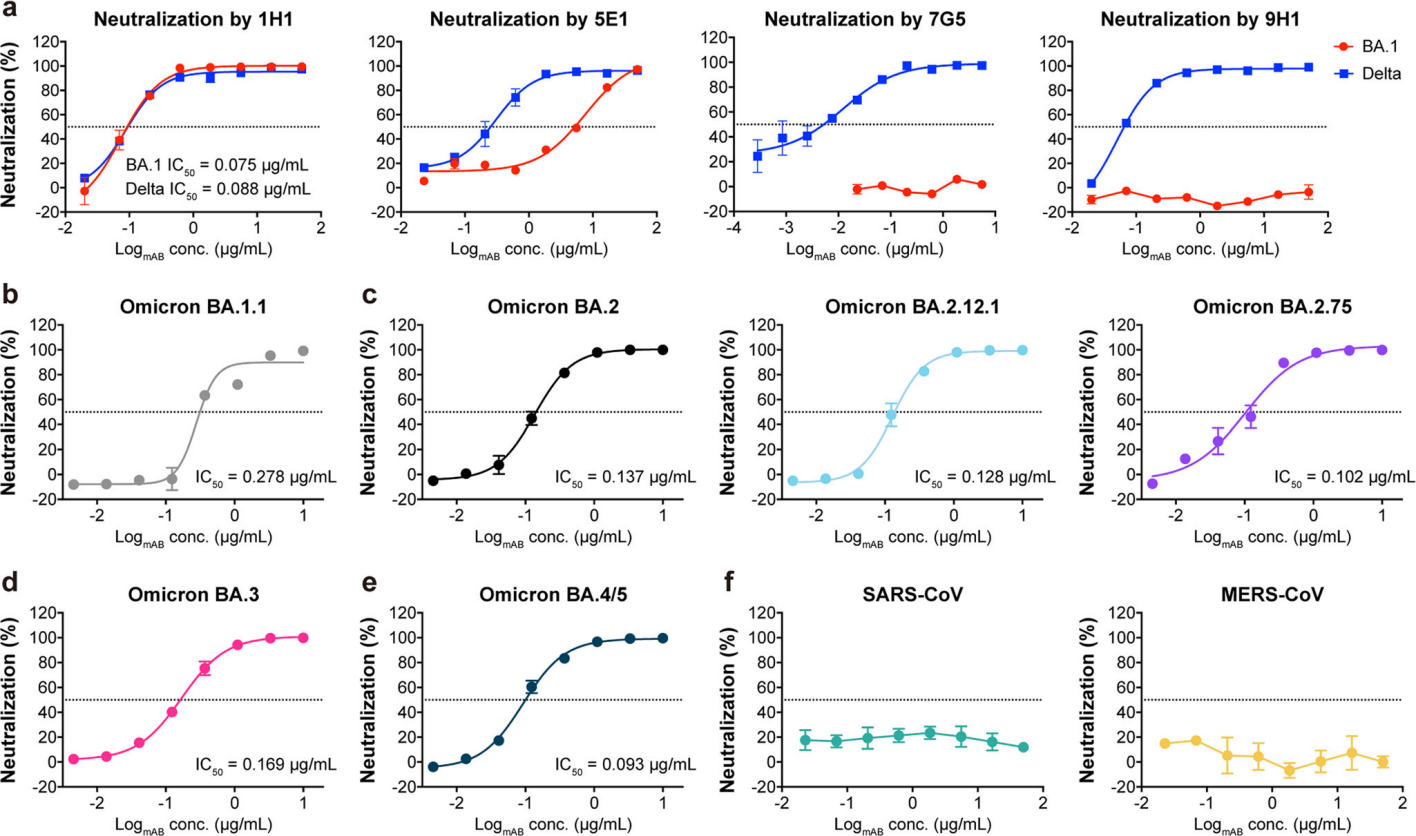
1H1 neutralizes SARS-CoV-2 sublineages. **a** neutralizing potency evaluation of 1H1, 5E1, 7G5 and 9H1 against pseudoviruses of Delta and Omicron BA.1. **b**–**g** neutralization of pseudotyped Omicron sublineages BA.1.1 (**b**), BA.2, BA.2.12.1 and BA.2.75 (**c**), BA.3 (**d**) and BA.4/5 (**e**) by 1H1. **f** neutralization of pseudotyped SARS-CoV (left) and MERS-CoV (right) by 1H1. Dashed line indicates a 50% reduction in viral neutralization. Data are shown as the mean of independent triplicates. Error bars indicate standard deviation (SD) of at least three biological replicates.

In a previous study, we used a single B cell SMab^®^ platform to generate a panel of RBD-binding RmAbs^35^. We identified four RmAbs that can potently neutralize the SARS-CoV-2 prototype strain, including 1H1, 5E1, 7G5 and 9H1. We further compared these four RmAbs for neutralization of the Delta and Omicron BA.1 pseudoviruses (Fig. 1a). All of these RmAbs neutralized the Delta variant, with comparable half-maximal inhibitory concentration (IC_50_) values in pseudovirus experiments (Fig. 1a). However, in the Omicron neutralization tests, RmAbs 5E1, 7G5 and 9H1 lost their neutralization activity against BA.1 variant (IC_50_ > 10 μg/mL) (Fig. 1a). By contrast, 1H1 remained highly effective against BA.1 with an IC_50_ value of 74.7 ng/mL, which was comparable to Delta (IC_50_ = 88.1 ng/mL) (Fig. 1a). Thus, only 1H1 retained neutralizing potency against BA.1.

We subsequently evaluated the neutralization activity of 1H1 with the pseudotyped Omicron sublineages BA.1.1, BA.2.12.1, BA.2.75, BA.3 and BA.4/5. Notably, 1H1 retained potency against all Omicron sublineages tested (Fig. 1b–e), especially BA.1, which was about 3~4-fold better than other Omicron sublineages (Fig. 1a). 1H1 showed a slightly reduced ability to neutralize other Omicron variants indicating that additional Omicron mutations may affect antibody potency. In addition, 1H1 was ineffective in neutralizing SARS-CoV and MERS-CoV (IC_50_ > 10 μg/mL) (Fig. 1f), suggesting its specificity to SARS-CoV-2. Taken together, 1H1 can neutralize SARS-CoV-2 variants, especially cross-reacting against different Omicron sublineages.

### The binding properties of 1H1 to spike and RBD proteins of the SARS-CoV-2 variants

**Fig. 2:**
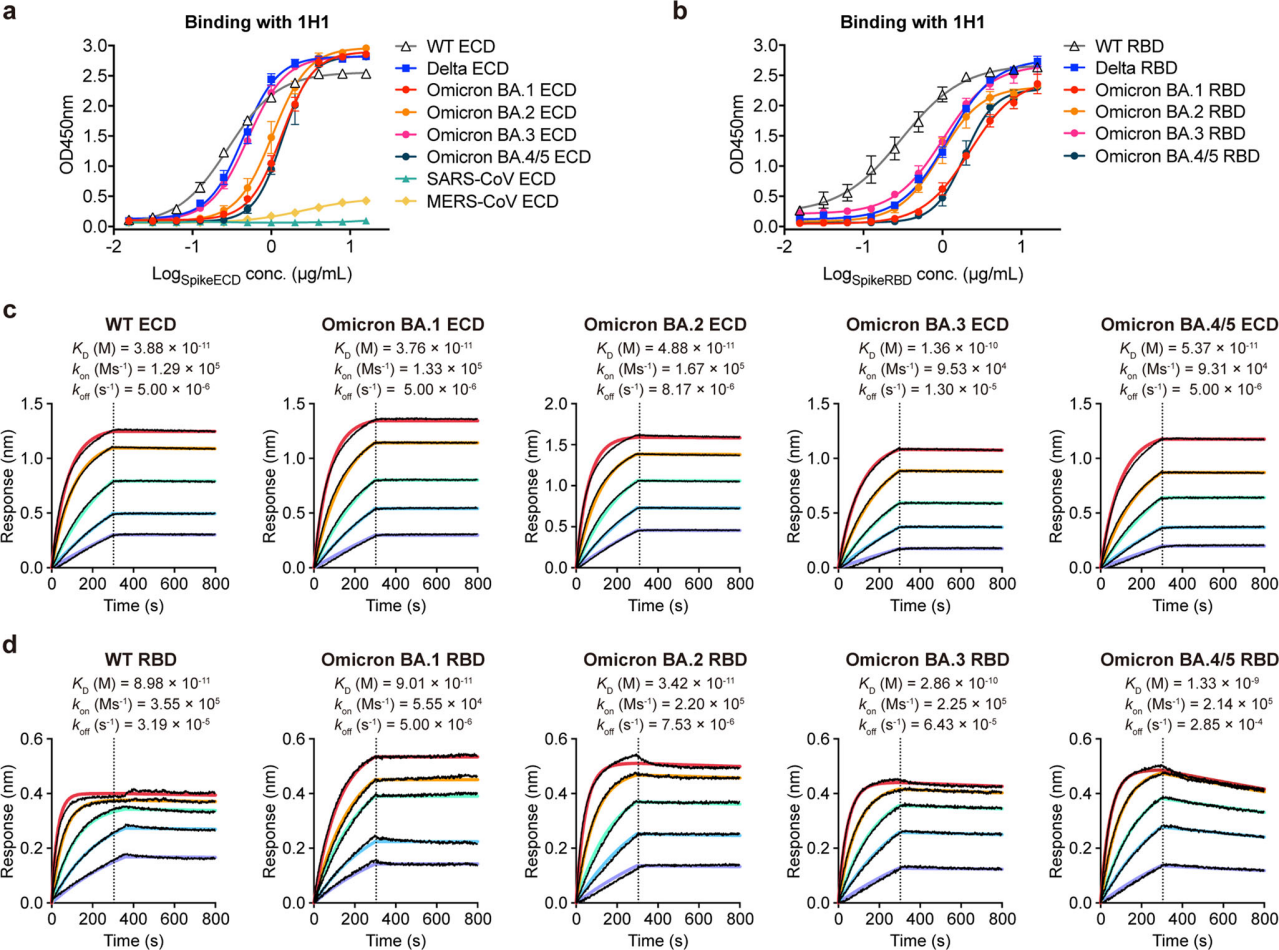
Broad binding affinity of 1H1. **a**, **b** binding of 1H1 to different spikes (**a**) or RBDs (**b**) of SARS-CoV-2 variants as measured by ELISA. Data are shown as the mean values of three replicates. Error bars indicate standard deviation (SD) of at least three biological replicates. **c** binding kinetics of 1H1 to different spike ECD proteins of SARS-CoV-2 variants as measured by BLI. **d** binding kinetics of 1H1 to different spike RBD proteins of SARS-CoV-2 variants as measured by BLI. Black lines were the original curves, while colored lines were the fitted curves. *K*_*D*_ apparent values are shown for 1H1 IgG binding to spike ECD and RBD proteins using a 1:1 global fit model.

Recent SARS-CoV-2 Omicron variants with newly occurred mutations on the spike protein have raised concerns on immune escape from antibody recognition^9,14,31^. We first determined the binding ability of 1H1 with different spike ECD proteins from SARS-CoV-2 variants using an enzyme-linked immunosorbent assay (ELISA). 1H1 was able to bind to all the spikes, indicating its broad binding ability (Fig. 2a). Two additional spike proteins from previously appeared coronaviruses SARS-CoV and MERS-CoV were also tested. Consistent with the neutralization experiments, 1H1 did not bind to SARS-CoV spike and MERS-CoV spike (Fig. 2a). In addition, we also evaluated the binding ability of 1H1 to different RBDs. The RBD binding ability of 1H1 was comparable to that of the spike ECD from SARS-CoV-2 variants, with slightly higher binding to WT RBD than to the RBDs of Delta and Omicron sublineages (Fig. 2b).

We then performed biolayer interferometry (BLI) experiments to evaluate binding affinities of 1H1 to spike ECDs from SARS-CoV-2 variants. 1H1 exhibited approximately similar binding affinities to SARS-CoV-2 WT and Omicron sublineage spikes, with values ranging from picomolar (3.88 × 10^−11^ M) to subnanomolar (1.36 × 10^−10^ M) (Fig. 2c). In contrast, the binding affinity of 1H1 to BA.4/5 RBD was comparatively decreased when compared to RBDs of other variants (Fig. 2d). When comparing RBDs and spike ECDs of Omicron sublineages, the binding affinities of 1H1 to each were similar, except for the reduced binding of 1H1 to Omicron BA.4/5 RBD, which was still at the nanomolar level (1.33 × 10^−9^ M) (Fig. 2c, d).

### Cryo-EM structure determination of Omicron BA.1 spike in complex with 1H1

**Fig. 3:**
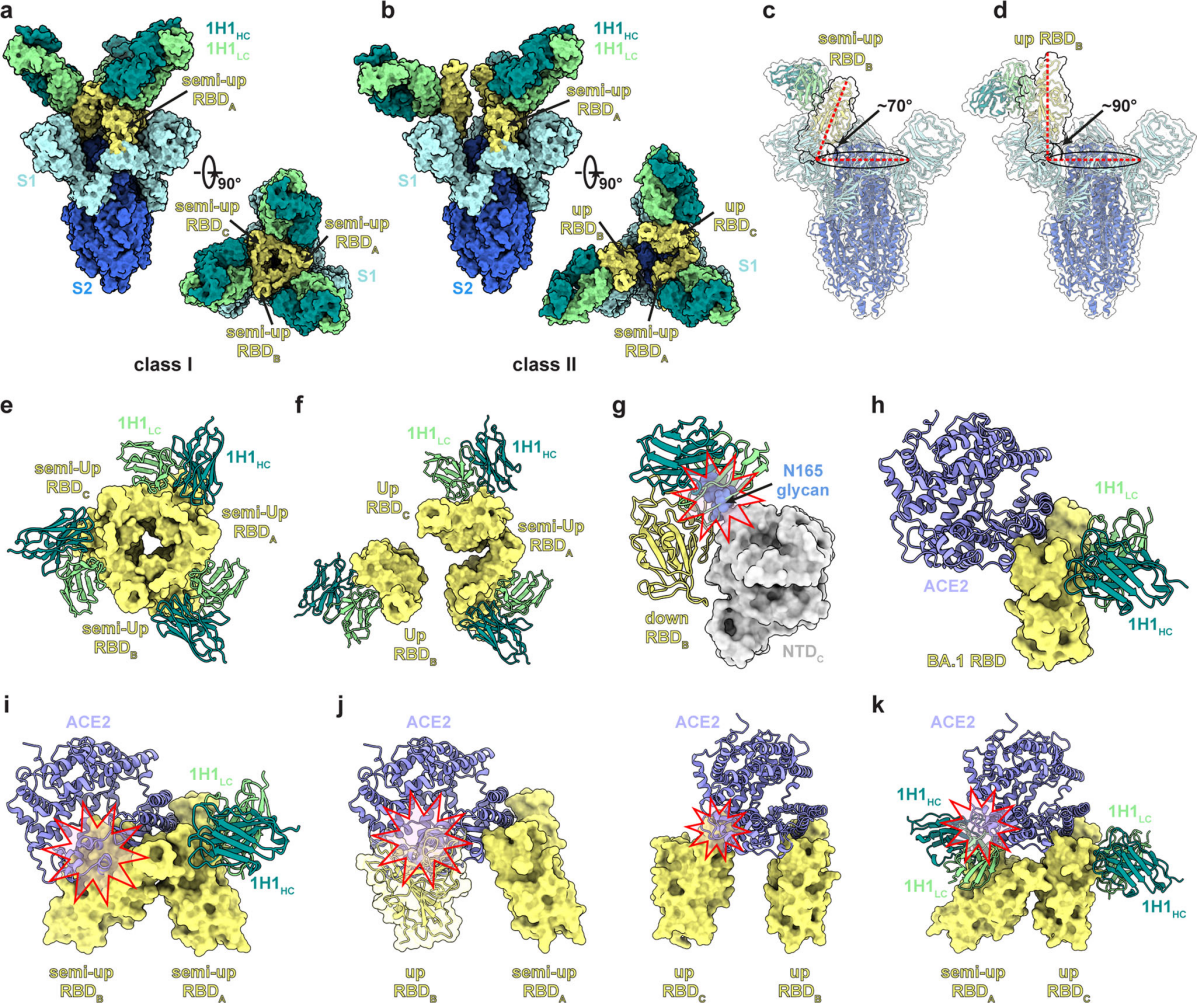
Cryo-EM structures of BA.1 spike protein in complex with 1H1 RmAbs. **a**, **b** the overall cryo-EM structures of the BA.1 spike-1H1 Fab complexes. **a** class I, 3.41 Å, revealing binding of 1H1 to RBDs in the “3 semi-up” state; **b** class II, 3.70 Å, revealing binding of 1H1 to RBDs in “1 semi-up/2 up” state. **c**, **d** the tilt angle of the semi-up (**c**) and up (**d**) BA.1 RBDs are defined by the angle between the long axis of RBD (red line) and its projection on the horizontal plane (black ellipse)^37^. **e** a close-up view of a 3-fold symmetric conformation with three 1H1 Fabs bound to the “3 semi-up” RBDs in class I. **f** a close-up view of an asymmetric conformation with three Fabs bound to the “1 semi-up/2 up” RBD conformation in class II. **g** superposition of the BA.1 RBD-1H1 Fab model to a down RBD of the spike trimer demonstrates that 1H1 cannot bind to a down RBD because of steric clashes by N165 glycan on the adjacent NTD. **h**, superposition of the local-refined RBD-ACE2 model to that of BA.1 RBD-1H1 Fab model shows no steric hindrance between 1H1 Fab and ACE2. **i** superposition of the local-refined RBD-ACE2 model to that of BA.1 semi-up RBD in class I shows a steric hindrance between ACE2 and an adjacent semi-up RBD. **j**, **k** superposition of the local-refined RBD-ACE2 model to that of BA.1 semi-up RBD_A_ (**j**, left), up RBD_B_ (**j**, right) and up RBD_C_ (**k**) in class II shows steric hindrance between ACE2 and an adjacent RBD or a 1H1 Fab bound on the adjacent RBD.

To understand the structural basis of the neutralizing mechanism for RmAb 1H1, we determined cryo-electron microscopy (cryo-EM) structures of the prefusion BA.1 spike trimer in complex with 1H1 using the six proline-stabilized (HexaPro) Omicron BA.1 spike ECD and the 1H1 Fab fragment (Supplementary Fig. 1a, b). 3D classification revealed two classes of BA.1 spike-1H1 Fab complexes in which each RBD was bound with one 1H1 Fab, representing a 3-Fab-per-trimer binding mode (Fig. 3a, b). We refined both classes to the overall resolution of 3.41 Å and 3.70 Å (Supplementary Fig. 1c, d), respectively. Because of the conformational dynamics of the 1H1-bound RBDs, we performed local refinement to improve the resolution of the RBD-1H1 interface (Supplementary Fig. 1b, e). The local-refined density map was used to build structural models along with the predicted 1H1 Fab structure to illustrate detailed interactions of amino acid residues, and only the variable heavy chain (VH) and variable light chain (VL) domains of the 1H1 Fab were built in our final models because of the flexible nature of the 1H1-bound RBDs (Supplementary Fig. 1f, g).

In the class I complex, the BA.1 spike trimer is in the semi-open form with all three RBDs adopting a semi-up position with a tilt angle of ~70 degrees (Fig. 3c), preserving a 3-fold symmetric conformation. In the class II complex, only one RBD of the BA.1 spike trimer adopts a similar semi-up position while the other two RBDs adopt an up position with a tilt angle of ~90 degrees (Fig. 3d). Interestingly, we did not find any BA.1 RBD in the down conformation (Fig. 3e, f). Superposition of the BA.1 RBD-1H1 Fab complex structure with the BA.1 spike containing RBDs in the down conformation (PDB ID: 7WS5) indicates that the 1H1 light chain is sterically hindered by the N165 glycan on the adjacent NTD^36^ (Fig. 3g). This may explain why the 1H1-bound RBDs do not exist in the down conformation.

In both classes, each semi-up or up RBD is decorated with one 1H1 Fab on the outer surface of the RBD spatially distinct from the RBM, resulting in an epitope that does not overlap with the ACE2 binding site. Superposition of the up RBDs with the structures of BA.1 RBD-1H1 Fab and RBD-ACE2 complexes reveals no overlap between the 1H1 Fab and ACE2 on the same up RBD^36^ (Fig. 3h), indicating that 1H1 cannot directly compete with ACE2 for RBD binding.

We further analyzed how 1H1 disrupts the binding of spike proteins to the receptor ACE2 to achieve neutralization. In class I, three 1H1 Fabs coordinate to stabilize all three RBDs in the semi-up conformation through RBD-RBD interactions (Fig. 3e). In this state, the receptor-blocking activities of 1H1 are straightforward because it inhibits receptor recognition by preventing full exposure of the ACE2 binding site, and a steric hindrance is formed through the adjacent semi-up RBD (Fig. 3i). Steric hindrances caused by the adjacent RBD are also present in class II (Fig. 3j). Moreover, while the adjacent semi-up RBD cannot prevent ACE2 binding to the up RBD in the class II complex, 1H1 bound to this semi-up RBD can still block ACE2 binding (Fig. 3k).

To confirm the accuracy of the 3-Fab-per-trimer binding mode of 1H1 to the BA.1 spike protein, we also carried out cryo-EM experiments using the entire IgG of 1H1 (Supplementary Fig. 2). The 3D reconstruction displayed a binding mode in which three 1H1 Fab fragments (from three 1H1 IgG molecules) were bound to the BA.1 spike protein (Supplementary Fig. 2a, b). We refined it to an overall resolution of 3.68 Å (Supplementary Fig. 2c). The BA.1 spike protein contains two RBDs in the “up” conformation and one in the “semi-up” conformation, which is consistent with the 1H1 Fab-BA.1 spike complex in the class II conformation (Supplementary Fig. 2d).

We also confirmed our structural analysis by competitive ELISA experiments. 1H1 significantly inhibited the binding of ACE2 to Omicron BA.1 spike ECD (Supplementary Fig. 3a), while it failed to efficiently block the interaction between ACE2 and Omicron BA.1 RBD (Supplementary Fig. 3b). Thus, although 1H1 does not directly compete with ACE2 for RBD binding, it can block ACE2 binding to the spike trimer through steric hindrances of its own or formed by inducing RBDs to stabilize in the semi-up conformation, thereby exerting a neutralizing effect.

### Structural basis for the potent and broad neutralization of 1H1

**Fig. 4:**
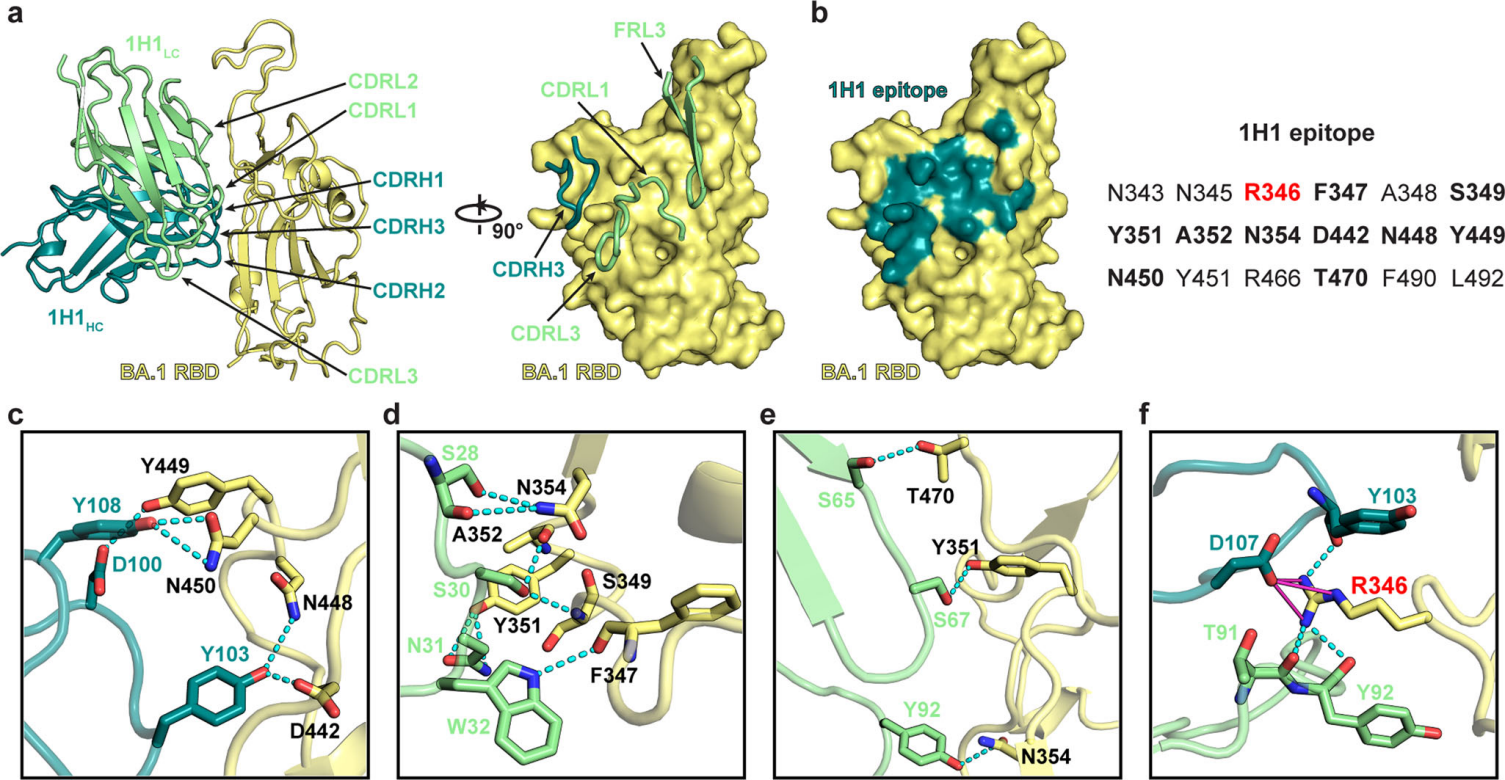
Structural details of interactions between the BA.1 RBD and 1H1 Fab for potent and broad neutralization. **a** overall structural model of BA.1 RBD in complex with 1H1 Fab and its CDRs are labeled. **b** the footprints of 1H1 are represented as surface and colored with dark cyan. The BA.1 RBD residues recognized by 1H1 are listed and a key interaction residue R346 of the BA.1 RBD is colored red. **c**–**f** the detailed interactions between the BA.1 RBD and 1H1 Fab including 1H1 heavy chain (**c**), 1H1 light chain (**d**, **e**) and the key residue R346-related interactions (**f**). The interacting residues of BA.1 RBD are shown as yellow sticks, the 1H1 heavy chain residues are shown as dark cyan sticks and the light chain residues are shown as light green sticks. Potential hydrogen bonds are represented as cyan dashed lines and salt bridges are represented by magenta lines, respectively.

To understand the structural basis of how 1H1 can broadly neutralize all the Omicron sublineages, further local refinement was applied to two adjacent semi-up RBDs of class I to a resolution of 3.52 Å, with one 1H1 Fab bound to each RBD. The improved local densities revealed detailed molecular interactions within the binding interface (Supplementary Fig. 1e). We utilized the BA.1 RBD-1H1 Fab interfaces for the following structural description and analysis. Similar to D2, a recently reported antibody with broad-spectrum neutralizing potency against Omicron sublineages, 1H1 also does not directly compete with ACE2 for RBD binding^37^. The 1H1 epitope is located on the outer surface of the RBD, overlapping with part of the RBD-4 and RBD-5 communities defined by previous research^38^ (Fig. 4a). When 1H1 binds to the BA.1 RBD, the 1H1 VL dominates most of the interactions with the BA.1 RBD, while the VH participates in part of the interactions (Fig. 4a). The interaction involves a buried surface area of 930 Å^2^ from 1H1 and 944 Å^2^ from the BA.1 RBD (Fig. 4b). Specifically, three of the complementarity-determining regions (CDRs; CDRL1, CDRL3 and CDRH3) of 1H1 directly participate in RBD binding (Fig. 4a), and the 1H1 light chain framework region 3 (FRL3) is also involved in the interactions (Fig. 4a).

Among 15 mutations found on the Omicron BA.1 RBD, the 1H1 epitope does not include any mutation found in BA.1 nor any additional mutation sites found in other Omicron sublineages including BA.2, BA.3, BA.4 and BA.5 (Fig. 4b). Therefore, these Omicron mutations will not directly affect the interactions between the RBD and 1H1. At the BA.1 RBD-1H1 interface, RBD residues R346, F347, S349, Y351, A352, N354, D442, N448, Y449, N450 and T470 have extensive interactions with 1H1 VH residues D100, Y103, D107, Y108 (Fig. 4c), and 1H1 VL residues S28, S30, F32, S65, S67, T91 and Y92 (Fig. 4d, e). Several hydrogen bonds and salt bridges are identified at the contact surface of BA.1 RBD and 1H1 Fab, representing the unique network associated with individual CDRs or FRs and amino acid residues within the epitope corresponding to 1H1. Notably, the BA.1 RBD residue R346 has extensive interactions with 1H1 VH residues Y103, G104, A105, P106 and D107, and VL residues W32, T91 and Y92, including hydrogen bonds between R346 and the VH Y103, the VL T91 and Y92, and salt bridges between R346 and the VH D107 (Fig. 4f). The R346K mutation is expected to disrupt these interactions, resulting in reduced neutralization against Omicron BA.1.1 compared to BA.1, BA.2 (including BA.2.12.1 and BA.2.75), BA.3 and BA.4/5 (Fig. 1b). Another significant mutation found in Omicron BA.4/5 is L452R, which does not affect 1H1 binding since L452 has no obvious interaction with 1H1. Instead, it may form a new hydrogen bond with VL T53 from 1H1, resulting in no significant change in the neutralization efficiency of 1H1 against BA.4/5 and Delta pseudoviruses compared to BA.1 (Fig. 1e). In addition, although the binding of 1H1 to the Omicron BA.4/5 RBD is significantly reduced (~100-fold) compared to the WT RBD, the affinity between them remains in the low nM range (~1.33 nM) (Fig. 2d). Therefore, we conclude that the strong binding ability of 1H1 and its distinct epitope that is not affected by Omicron mutations make 1H1 a potential high potency, broad-spectrum neutralizing mAb against different SARS-CoV-2 VOCs, including Delta and Omicron sublineages.

## Discussion

Since the early COVID-19 pandemic, a large number of potent neutralizing antibodies against SARS-CoV-2 have been reported^27,35–37,39–42^. However, the continuous emergence of SARS-CoV-2 variants, especially the Omicron sublineages, with high levels of mutations has led to serious concerns about immune evasion, vaccine failure, and the lack of effective neutralizing antibodies for clinical treatment^2,3,6,8,9,11,33,43^. Thus, there is an urgent need to develop new antibodies with broad-spectrum neutralizing activity against Omicron variants.

Many recent studies have reported a significant reduction or complete loss of activity against Omicron as well as its sublineages by a large number of neutralizing antibodies^2,3,6,8,9,11,12,14,33^, and only a few antibodies with broadly neutralizing activity have been reported. Interestingly, although many studies have shown that antibodies targeting RBM may easily lose their neutralizing potency due to the high mutation rate in the RBM region, recent studies have reported several ACE2-blocking antibodies with broad-spectrum neutralizing ability, including S2K146^7,44^, 87G7^45^ and F61^37^ that directly target RBM, and LY-CoV1404^46^ whose epitope slightly overlaps with the ACE2 binding site. Here we identified a highly potent and broad-spectrum neutralizing RmAb 1H1 that targets outside of the RBM against different SARS-CoV-2 VOCs including Delta and Omicron sublineages.

In our previous study, 1H1 showed potent neutralizing effects against SARS-CoV-2 WT, Alpha, Beta, Gamma, Epsilon and Iota variants in pseudovirus experiments^35^. We further demonstrated that 1H1 was also highly potent against Delta and Omicron BA.1, BA.1.1, BA.2, BA.2.12.1, BA.2.75, BA.3 and BA.4/5 pseudoviruses with IC_50_ values below ~150 ng/mL. The binding kinetics also indicated that 1H1 has similar binding affinities to spike ECD and RBD of SARS-CoV-2 WT and Omicron sublineages in the picomolar to the subnanomolar range, except that the binding of 1H1 to Omicron BA.4/5 RBD was slightly reduced. Furthermore, we performed cryo-EM structural analyses of the prefusion BA.1 spike trimer in complex with 1H1 Fabs and IgGs. This indicated that 1H1 could bind to a conserved region of the BA.1 RBD, avoiding mutation sites of almost all major circulating Omicron variants (except the R346K in BA.1.1 and R346T in BQ.1.1, XBB.1 and XBB.1.5). Pseudovirus neutralization experiments further confirmed our structural analysis that the neutralization potency of 1H1 against Omicron BA.1.1 was reduced compared with BA.1, BA.2, BA.3 and BA.4/5.

The 1H1 epitope is similar to two recently reported broadly neutralizing antibodies, COVOX-58^8^ and D2^37^, which partially overlap with the epitope of the typical Class 3 antibody S309^47^. All of these four antibodies have non-overlapping epitopes with ACE2 and can bind to the RBDs in different conformations. While S309 recognizes a highly conserved epitope on the RBD that comprises the N343-linked glycans, the 1H1 epitope neither overlaps with the ACE2 binding site nor interacts with the N343-linked glycans (Supplementary Fig. 4a). Like COVOX-58 and D2, the major neutralizing mechanism of 1H1 would not be direct ACE2 competition. The similar binding modes of these three antibodies indicate that they are unaffected by most Omicron mutations due to binding to a conserved region of RBD and maintaining potent neutralizing activity^8,37^ (Supplementary Fig. 4b). Besides similarities, 1H1 stands out with its distinct spatial arrangement of its VH and VL that is just opposite to that of COVOX-58 and D2 (Supplementary Fig. 4a). 1H1 mainly interacts with BA.1 RBD through the VL and CDRH3 (Fig. 4a and Supplementary Fig. 4a). Notably, the epitope of 1H1 was found to be highly conserved among all the Omicron variants of high frequencies (Supplementary Fig. 5a, b), not only among ancestral Omicron sublineages but also the recently emerging Omicron variants (Supplementary Fig. 5c)^48–51^. In comparison to COVOX-58 and D2, 1H1 possesses the unique ability to resist the L452 mutation, allowing it to maintain its powerful neutralizing activity against SARS-CoV-2 variants that have the L452R substitution on the spike protein, including Delta, BA.4/5, BQ.1 and BQ.1.1. This superiority makes 1H1 a promising solution against the rapidly evolving virus. Additionally, this gives 1H1 a broader spectrum of neutralizing activity against SARS-CoV-2 variants, further increasing its potential as a valuable tool to fight against the virus.

In summary, we characterize a potent neutralizing RmAb 1H1 that has broad-spectrum neutralizing ability against most of the current global-circulating Omicron sublineages. The newly characterized neutralizing potential of 1H1 may provide a new approach for developing multifunctional, cost-effective therapeutics and point-of-care diagnosis. Moreover, it can also be used as a typical antibody model to design broad-spectrum neutralizing antibodies against new SARS-CoV-2 variants that may emerge in the future.

## Methods

### Expression and purification of SARS-CoV-2 Omicron spike protein

Soluble 6P-stabilized SARS-CoV-2 spike proteins (WT, BA.1, BA.2, BA.3 and BA.4/5) were expressed by transient transfection^20,36^. In brief, the genes encoding spike ECD of different variants were synthesized and codon-optimized by GenScript, and then cloned into the mammalian expression vector pcDNA3.1 (Invitrogen). The plasmid was transfected using PEI into FreeStyle 293-F cells (Invitrogen). Transfected cells were cultured at 35 °C, 8% CO_2_, and the cell culture supernatant was collected following 4 to 5 days of incubations. Protein was purified from filtered cell supernatants using Ni Sepharose resin (Cytiva) and further purified by gel filtration chromatography using a Superose 6 10/300 column (Cytiva) in 1 × TBS (20 mM Tris-HCl, 200 mM NaCl, pH8.0).

### Generations of rabbit monoclonal antibodies against SARS-COV-2 spike proteins

We have complied with all relevant ethical regulations for animal testing and research. One-month-old female New Zealand Big White Rabbits (Yurogen, Wuhan, China) were utilized for this study. The research protocol was approved by the Ethics Committee from Nanjing Drum Tower Hospital Institution Animal Care and Use Committee (IACUC) (protocol No. 2020AE01120). RmAbs 1H1, 5E1, 7G5 and 9H1 were generated using the SMab platform from Yurogen Biosystems^35^. Briefly, rabbits were immunized with DNA vaccines encoding SARS-COV-2 RBDs 3 times followed by 2 boosts with recombinant SARS-COV-2 S1 proteins. Rabbit bleed titers were monitored by ELISA against spike ECD protein, S1 protein, RBD protein or RBD variants proteins. Splenocytes from rabbits with the best overall titers against these proteins were prepared and used for single B cell sorting. Primary B cell culture supernatants were screened against RBD or S1 by direct ELISA. IgG variable region from those ELISA-positive clones was amplified by RT-PCR. Successfully recovered IgG variable regions were assembled into full-length IgG with mammalian expression components for transient IgG expression in HEK293T cell lines. The cultured supernatants were screened again by ELISA. IgG variable regions from a positive clone at this step were cloned into pcDNA3.4 vector for recombinant monoclonal antibody expression in HEK293F and the recombinant antibodies were purified by protein A affinity chromatography. Identified RmAbs were further evaluated by pseudovirus and live virus neutralization assays and ACE2 receptor blocking ELISA assay.

### Production of Fab fragments from rabbit monoclonal antibodies

Rabbit monoclonal antibodies were buffer exchanged to the 20 mM PBS, 10 mM EDTA, pH 7.0, and concentrated to 4 mg/mL using centrifuge filters (Merck, Cat: UFC803096). Then antibodies were fragmented using immobilized papain according to the protocol (ThermoFisher, Cat: 20341). Briefly, to prepare the papain resin for use, it was removed from the storage buffer, mixed with the 4 ml activation buffer (20 mM PBS, 10 mM EDTA, 20 mM Cysteine-HCl, pH 7.0), incubated at room temperature for 10 mins. Next, the papain resin was centrifuged at 1000 g for 5 min, and the supernatant was discarded. The papain resin was finally re-suspended in 0.5 mL activation buffer.

Then the immobilized papain resin and buffer-exchanged antibody solution were mixed and placed in a 37 °C incubator for 5 h at 180 rpm. After the reaction, the fragmented antibody sample was separated from immobilized papain resin using Pierce™ Centrifuge Columns. The resin-bound enzyme was kept above the column and the antibody fragments were collected in the flow-through fraction. Fab and Fc mixtures were further incubated with immobilized rProteinA Beads 4FF (smart-lifesciences, Cat: SA012200) at room temperature for 2 h. The Fab fragments were buffer exchanged into PBS buffer and confirmed by the SDS-PAGE.

### SARS-CoV-2 pseudovirus neutralization assays

Pseudovirus neutralization assays were performed following the conditions and methods outlined in our previous study as a ref. ^52–54^. Briefly, series diluted RmAbs were incubated with 2 × 10^3^ TCID_50_ SARS-CoV-2 pseudoviruses including WT, Delta and Omicron sublineages separately for an hour at 37 °C. 100 μL of freshly trypsinized HEK293T-ACE2 cells (2 × 10^4^ cells/well) were added to 96-well plates. After 48 h of incubation at 37 °C, the luminescence was measured using the Bio-lite Luciferase assay system (Cat# DD1201-01, Vazyme, Nanjing, China) and detected for relative light units (RLUs) via Spark multimode microplate reader (Tecan, Männedorf, Switzerland). Half-maximum inhibitory concentration (IC_50_) was calculated by the concentration of RmAb at which RLUs were reduced by 50% compared to viral control wells, while background RLUs of cell control wells were subtracted.

### ELISA experiments

Serially diluted spike ECD and RBD proteins from SARS-CoV-2 WT, Delta, Omicron BA.1, BA.2, BA.3 and BA.4/5 were added to the ELISA 96-well plates (BIOFIL) (100 μL/well) at 4 °C overnight. Plates were blocked with blocking buffer (5% skim milk powder in PBS, 0.05% Tween-20 (PBST)) at 37 °C for 1 h. 30 ng/well of the 1H1 mAbs were added to the plates and incubated at 37 °C for 2 h. After washing with PBST, the horseradish peroxidase (HRP) Goat anti-rabbit IgG (H + L) (ABclonal, AS014, diluted 1:5000) at 37 °C for 1 h. Lastly, the TMB substrate (Beyotime) was added, and absorbance was measured at 450 nm by a microplate reader (SpectraMax M4).

### Bio-layer interferometry assays

The binding kinetics of 1H1 to spike or RBD proteins from SARS-CoV-2 variants was performed by biolayer interferometry analysis on the GatorPrime Label-Free Bioanalysis instrument (Gator Bio, Palo Alto, CA, USA). Briefly, the protein A probes (Gator Bio) were prewetted in Q buffer in advance to be balanced. Subsequently, 1 nM of 1H1 mAbs were immobilized to the probes. Then the immobilized probes were immersed in the solution containing two-fold serial diluted spike or RBD proteins. The 1H1-loaded probes were incubated with various antigen proteins for 300 s in the sample well during the association step and then followed by 600 s probe incubation in Q buffer for the dissociation step.

### Cryo-EM sample preparation and data collection

Purified SARS-CoV-2 Omicron BA.1 spike protein was diluted to a concentration of 1.6 or 0.8 mg/mL in PBS, pH 7.4, and was incubated with 1H1 Fab or IgG at a molar ratio of 1:3 or 1:1.5, respectively. To prevent aggregation during vitrification, 0.01% (w/v) n-dodecyl β-D-maltoside (DDM) was added to the sample before plunge freezing. The mixture sample (3 μl) was applied onto an H_2_/O_2_ glow-discharged, 300-mesh R1.2/1.3 copper grid (Fab) or R0.6/1 gold grid (IgG) (Quantifoil), respectively. The grid was then blotted for 2.5 s with a blot force of −1 at 8 °C and 100% humidity and plunge-frozen in liquid ethane using a Vitrobot (ThermoFisher Scientific). Cryo-EM datasets were collected at a 300 kV Titan Krios microscope (ThermoFisher Scientific) equipped with a K3 detector (Gatan). For the BA.1 spike-1H1 Fab dataset, the exposure time was set to 2.0 s with a total accumulated dose of 60 electrons per Å^2^, which yields a final pixel size of 0.832 Å. For the BA.1 spike-1H1 IgG dataset, the exposure time was set to 2.4 s with a total accumulated dose of 60 electrons per Å^2^, which yields a final pixel size of 0.82 Å. 3177 micrographs of BA.1 spike-1H1 Fab complex and 2642 micrographs of BA.1 spike-1H1 IgG complex were collected with a defocus range comprised between 1.2 and 2.5 μm using SerialEM^55^.

The statistics of cryo-EM data collection are summarized in Table 1.

**Table 1:** Cryo-EM data collection, refinement and validation statistics.

	BA.1 spike-1H1 Fab class I (EMDB-34407) (PDB 8H00)	BA.1 spike-1H1 Fab class II (EMDB-34408) (PDB 8H01)	BA.1 spike-1H1 Fab Local refine (EMDB-34406) (PDB 8GZZ)	BA.1 spike-1H1 IgG (EMDB-35328) (PDB 8ITU)
Data collection and processing				
Magnification	59,000×	59,000×	59,000×	29,000×
Voltage (kV)	300	300	300	300
Electron exposure (e–/Å^2^)	60	60	60	60
Defocus range (μm)	−1.2 to −2.5	−1.2 to −2.5	−1.2 to −2.5	−1.2 to −2.5
Pixel size (Å)	0.832	0.832	0.832	0.82
Symmetry imposed	C3	C1	C3	C1
Initial particle images (no.)	235,203	235,203	235,203	204,370
Final particle images (no.)	69,259	89,494	69,259	130,665
Map resolution (Å)	3.41	3.70	3.52	3.68
FSC threshold	0.143	0.143	0.143	0.143
Map resolution range (Å)	3.41–6	3.70–8	3.62–6	3.68–8
Refinement				
Initial model used (PDB code)	7WS5	7WS5	7WS6	8H01
Model resolution (Å)	3.41	3.70	3.52	3.68
FSC threshold	0.143	0.143	0.143	0.143
Model resolution range (Å)	3.41	3.70	3.52	3.68
Map sharpening *B* factor (Å^2^)	−113	−102	−117	−121
Model composition				
Non-hydrogen atoms	30,181	30,181	6568	30,144
Protein residues	3803	3803	854	3803
Ligands	45	45	0	44
*B* factors (Å^2^)				
Protein	89.05	165.66	75.84	165.64
Ligand	91.08	138.69	N/A	136.17
R.m.s. deviations				
Bond lengths (Å)	0.004	0.003	0.004	0.004
Bond angles (°)	0.575	0.538	0.620	0.939
Validation				
MolProbity score	1.66	1.76	1.81	1.77
Clash score	7.67	9.26	8.56	9.38
Poor rotamers (%)	0.00	0.00	0.00	0.00
Ramachandran plot				
Favored (%)	96.36	96.07	95.01	96.07
Allowed (%)	3.56	3.83	4.99	3.80
Disallowed (%)	0.08	0.11	0.00	0.13

### Cryo-EM data processing

All dose-fractioned images were motion-corrected and dose-weighted by MotionCorr2 software^56^ and their contrast transfer functions were estimated by cryoSPARC patch CTF estimation^57^. The following particle picking, extraction, 2D classification, Ab-Initio reconstruction, 3D classification, 3D refinements and local resolution estimation were all carried out in cryoSPARC. For the BA.1 spike-1H1 Fab dataset, the final 3D reconstructions were obtained using non-uniform refinement, achieving a resolution of 3.41 Å for the semi-open conformation (class I) with C3 symmetry, and 3.70 Å for the ‘1 semi-up/2 up’ conformation (class II) with C1 symmetry. To improve the resolution for the binding interface, a local refinement focusing on the BA.1 RBD-1H1 variable domain region was carried out, achieving a 3.52 Å map representing the RBD-1H1 interface. For the BA.1 spike-1H1 IgG dataset, the final 3D reconstructions were obtained using non-uniform refinement, achieving a resolution of 3.68 Å.

The full cryo-EM data processing workflow is described in Supplementary Fig. 1 and 2.

### Model building and refinement

To build the structures of the SARS-CoV-2 Omicron BA.1 spike-1H1 Fab complexes, the recently reported structural model of the BA.1 spike glycoprotein in complex with the 510A5 neutralizing antibody Fab fragment^36^ (PDB: 7WS5) was placed and rigid-body fitted into the cryo-EM electron density maps using UCSF Chimera^58^ to build the BA.1 spike trimer. The 1H1 Fab model was predicted using Phyre2^59^. The manual and automated model building were iteratively performed using Coot 0.9.6^60^ and real-space refinement in Phenix 1.20^61^.

The data validation statistics are summarized in Table 1.

### ACE2 receptor blocking assay

Half maximal effective concentration (EC_50_) of Omicron spike or RBD protein binding to ACE2 was determined by ELISA assay. To determine whether ACE2 could compete with 1H1 bound to Omicron spike or RBD, the competitive ELISA assay was performed. Briefly, the ELISA plate was coated with 1 μg/ml recombinant ACE2 (Kactus Biosystems, Cat. No. ACE-HM501) overnight at 4 °C. 1H1 was serially diluted 3-fold starting from 3 μg/mL, and incubated with biotinylated Omicron BA.1 spike or RBD proteins at the concentration of EC_50_ at room temperature. After 1 h of incubation, the mixture of spike or RBD protein and 1H1 was then applied to ACE2-coated ELISA plates and incubated for 1 h. The biotinylated spike was detected via neutravidin conjugated to HRP and the RBD with mouse Fc tag was detected via goat anti-mouse IgG conjugated to HRP. Finally, ELISA plates were read at optical density (OD) of 450 nm and 630 nm with an Epoch microplate spectrophotometer (Biotek, USA).

### Statistics and reproducibility

The pseudovirus neutralization data from at least three biological replicates were analyzed in Prism 9 software (GraphPad) using a four-parameter logistic regression model. The bio-layer interferometry assays were performed in triplicates. The experiments are reproducible. Data are shown as mean ± SD. The resolution estimations of cryo-EM density maps are based on the 0.143 Fourier Shell Correlation (FSC) criterion.

### Reporting summary

Further information on research design is available in the Nature Portfolio Reporting Summary linked to this article.

## Supplementary information

Supplementary Information

Description of Additional Supplementary Data

Supplementary Data 1

Reporting Summary

## Data Availability

The source data for the graphs and charts in the figures is available as Supplementary Data 1. The coordinates and EM map files for the BA.1 spike-1H1 Fab class I complex, BA.1 spike-1H1 Fab class II complex and BA.1 RBD-1H1 Fab local-refined complex have been deposited in the Protein Data Bank (PDB) and the EM Data Bank (EMDB) under accession number PDB-8H00, PDB-8H01 and PDB-8GZZ, and EMDB-34407, EMDB-34408 and EMDB-34406, respectively. The coordinate and EM map file for the BA.1 spike-1H1 IgG complex have been deposited in the PDB and EMDB under accession number PDB-8ITU and EMDB-35328. For materials requests, please reach out to the corresponding authors.
