# Supplementary material for: Mechanism of a rabbit monoclonal antibody broadly neutralizing SARS-CoV-2 variants
Source: Commun Biol. 2023 Apr 3;6:364. doi: 10.1038/s42003-023-04759-5 (PMC10069731; doi:10.1038/s42003-023-04759-5)
Supplement: Supplementary file 1 — Supplementary Information [file 42003_2023_4759_MOESM1_ESM.pdf]

Supplementary Information for

Mechanism of a rabbit monoclonal antibody broadly neutralizing SARS-CoV-2 variants

Hangtian Guo[1,*], Yixuan Yang[1,*], Tiantian Zhao[2,*], Yuchi Lu[3,*], Yan Gao[3,4], Tinghan Li[1], Hang Xiao[5], Xiaoyu Chu[1], Le Zheng[1], Wanting Li[6], Hao Cheng[5], Haibin Huang[5], Yang Liu[5], Yang Lou[5], Henry C. Nguyen[3,4], Chao Wu[2,7], Yuxin Chen[8]†, Haitao Yang[3,4] and Xiaoyun Ji[1,7,8]†

[1]The State Key Laboratory of Pharmaceutical Biotechnology, School of Life Sciences, Nanjing University, Nanjing, Jiangsu 210023, China.

[2]Department of Infectious Diseases, Nanjing Drum Tower Hospital Clinical College of Nanjing University of Chinese Medicine, Nanjing, Jiangsu 210008, China.

[3]Shanghai Institute for Advanced Immunochemical Studies and School of Life Science and Technology, ShanghaiTech University, Shanghai 201210, China.

[4]Shanghai Clinical Research and Trial Center, Shanghai 201210, China.

[5]Yurogen Biosystem LLC, Wuhan, Hubei 430075, China.

[6]Department of Infectious Diseases, Nanjing Drum Tower Hospital Clinical College of Xuzhou Medical University, Nanjing, Jiangsu 210008, China.

[7]Institute of Viruses and Infectious Diseases, Nanjing University, Nanjing, Jiangsu 210008, China.

[8]Department of Laboratory Medicine, Nanjing Drum Tower Hospital, Nanjing University Medical School, Nanjing, Jiangsu 210008, China.

[*]These authors contributed equally: Hangtian Guo, Yixuan Yang, Tiantian Zhao, Yuchi Lu.

†To whom correspondence should be addressed. Tel: +86 025 89681656; Fax: +86 025 89681656; E-mail: yuxin.chen@nju.edu.cn (Y.C.), xiaoyun.ji@nju.edu.cn (X.J.);

This file includes:

Supplementary Figures 1–5

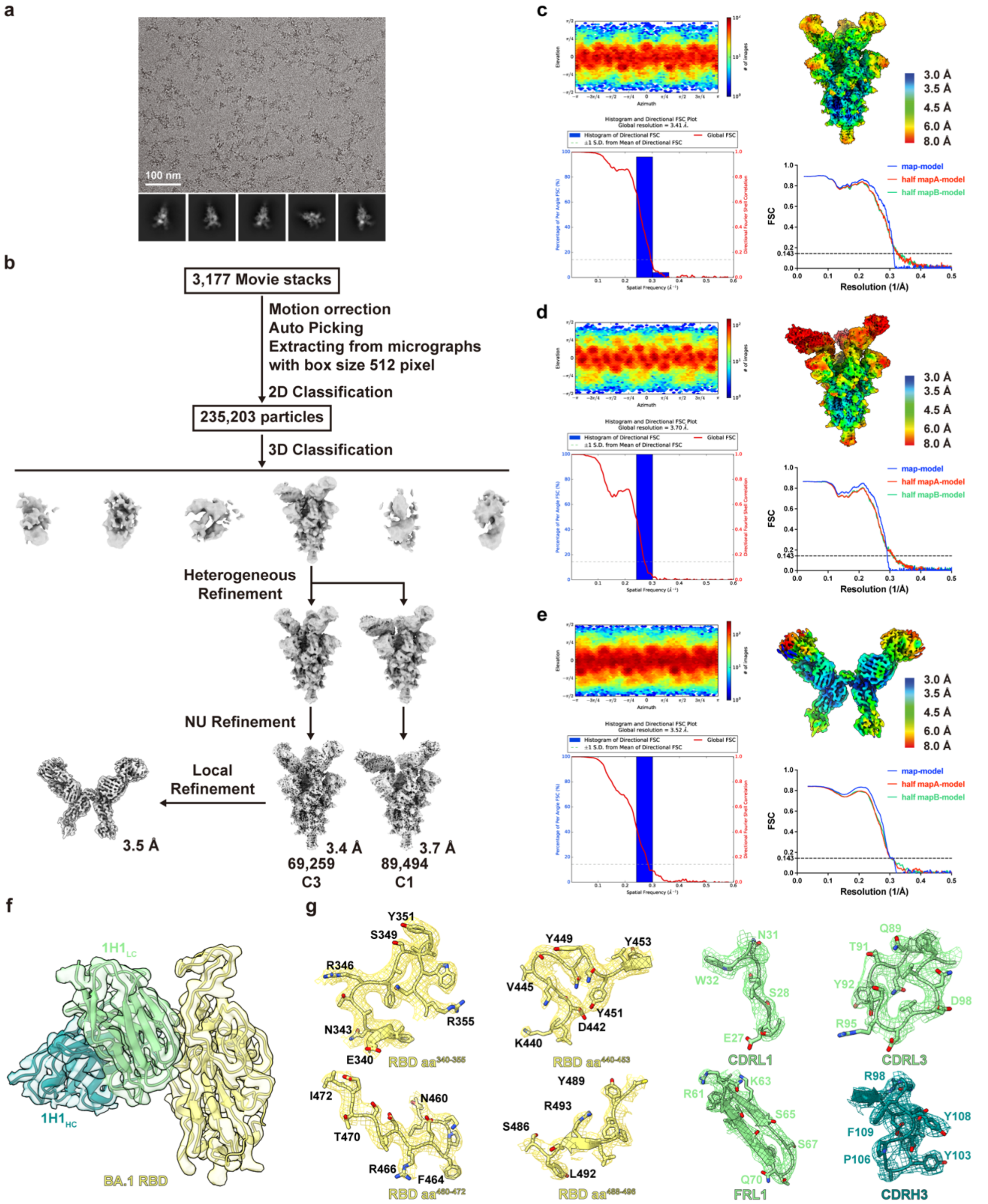

Supplementary Fig. 1 Cryo-EM data processing and validation for the SARS-CoV-2 Omicron BA.1 spike protein in complex with 1H1 Fab. a representative cryo-EM micrograph and 2D classes. **b** workflow for BA.1 spike-1H1 Fab 3D Reconstructions. **c-e**

the viewing direction distribution plot, global FSC and histogram, cryo-EM density colored according to the local resolution and model-map FSC for the class I (**c**), class II (**d**) and local refinement (**e**), respectively. **f** model-map fitting of the local-refined BA.1RBD-1H1 structures. **g** sharpened cryo-EM density maps (BA.1 RBD and 1H1 Fab interaction regions) are rendered as mesh with the corresponding models shown as sticks.

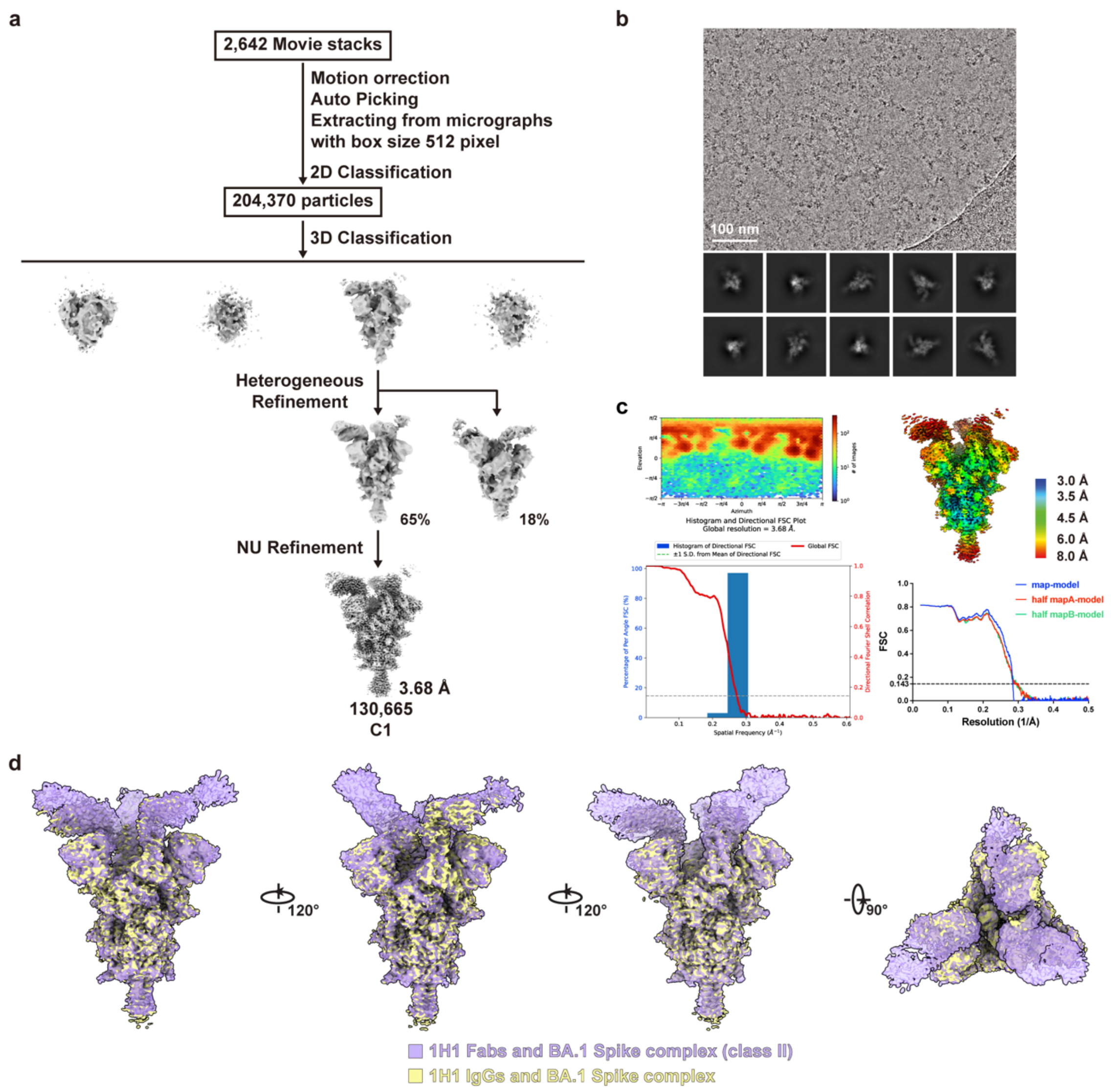

Supplementary Fig. 2 Cryo-EM data processing and validation for the SARS-CoV-2 Omicron BA.1 spike protein in complex with 1H1 IgG. a workflow for BA.1 spike-1H1 IgG 3D Reconstructions. **b** representative cryo-EM micrograph and 2D classes. **c** the viewing direction distribution plot, global FSC and histogram, cryo-EM density colored according to the local resolution and model-map FSC for the 1H1 IgG dataset. **d** comparison of the electron density maps between BA.1 spike-1H1 IgGs complex (yellow) and BA.1 spike-1H1 Fabs complex (class II, purple).

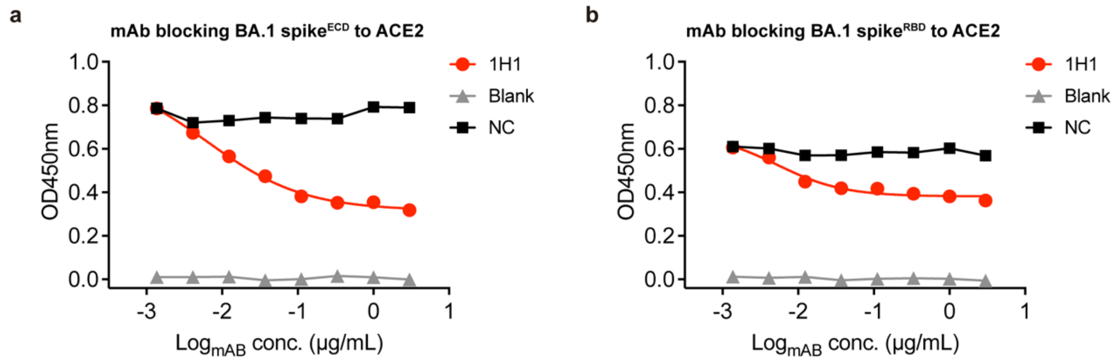

Supplementary Fig. 3 Competing ELISA assays. a, b the ability of 1H1 to compete with ACE2 for binding to Omicron BA.1 spike ECD (**a**) and RBD (**b**). The competition capacity of 1H1 was indicated by the level of reduction in the response unit of ACE2 compared with or without the addition of 1H1. Error bars indicate standard deviation (SD) of at least two biological replicates.

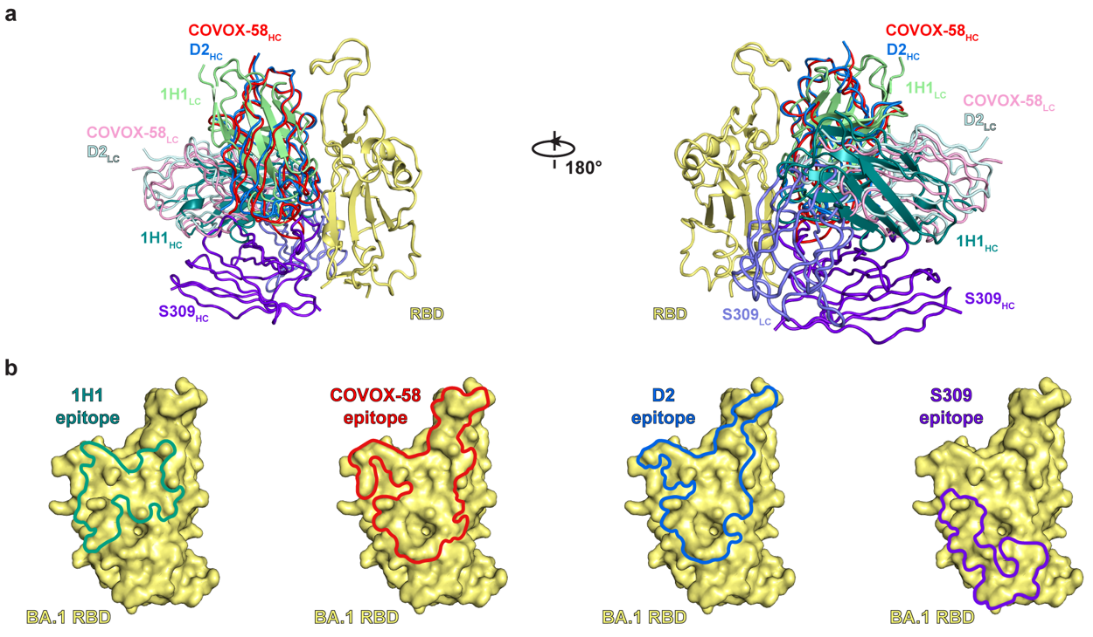

Supplementary Fig. 4 Comparison of 1H1 with COVOX-58, D2 and S309. a structural comparison of the RBD-1H1 and RBD-COVOX-58 (PDB ID: 7QNY), RBD-D2 (PDB ID: 7XMZ) and RBD-S309 (PDB ID: 7XCO) complexes. Superposition of 1H1, COVOX-58 and D2 shows a similar binding mode, where COVOX-58 and D2 share a nearly identical epitope and the epitope of 1H1 is slightly different from the other two. **b** the footprints of 1H1, COVOX-58, D2 and S309, respectively.

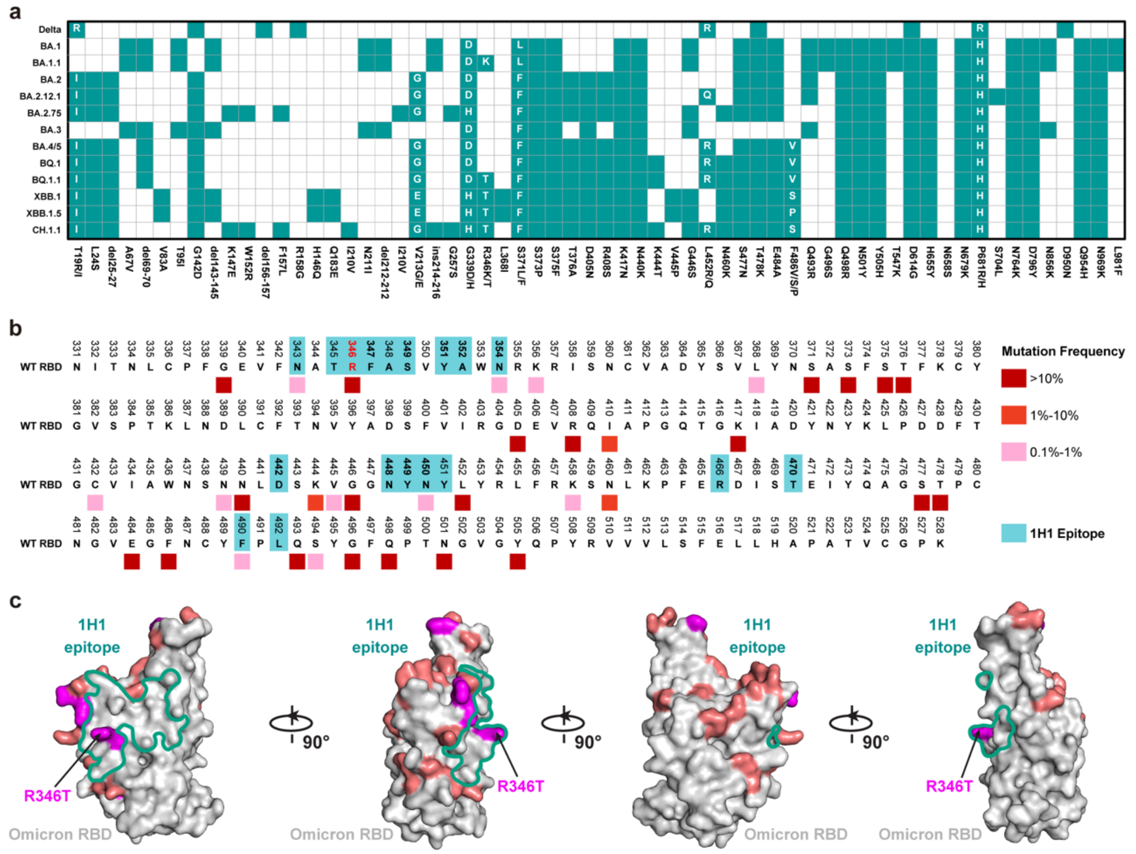

Supplementary Fig. 5 Mutations on the spike glycoprotein and illustration of 1H1 epitopes on RBD. a green grids indicate the corresponding PANGO lineage carries the corresponding mutation. **b** reported mutations in RBD from public database (https://ngdc.cncb.ac.cn/ncov/variation/spike) were marked by squares with different color. Residues involved in the binding to 1H1 were highlighted in blue. **c** Mutations found in ancestral Omicron variants (BA.1, BA.1.1, BA.2, BA.3 and BA.4/5) were highlighted in red and mutations found in recently emerging Omicron variants (BQ.1, BQ.1.1, XBB and XBB.1.5) were highlighted in magenta. The RBD was represented as gray surface.
