# Supplementary material for: Mechanism of a rabbit monoclonal antibody broadly neutralizing SARS-CoV-2 variants
Source: Commun Biol. 2023 Apr 3;6:364. doi: 10.1038/s42003-023-04759-5 (PMC10069731; doi:10.1038/s42003-023-04759-5)
Supplement: Supplementary file 4 — Reporting Summary [file 42003_2023_4759_MOESM4_ESM.pdf]

Corresponding author(s): Xiaoyun Ji

Last updated by author(s): Mar 23, 2023

Reporting Summary

Nature Portfolio wishes to improve the reproducibility of the work that we publish. This form provides structure for consistency and transparency in reporting. For further information on Nature Portfolio policies, see our Editorial Policies and the Editorial Policy Checklist.

Statistics

For all statistical analyses, confirm that the following items are present in the figure legend, table legend, main text, or Methods section.

n/a	Confirmed	
☐	☒	The exact sample size (n) for each experimental group/condition, given as a discrete number and unit of measurement
☐	☒	A statement on whether measurements were taken from distinct samples or whether the same sample was measured repeatedly
☒	☐	The statistical test(s) used AND whether they are one- or two-sided<br>*Only common tests should be described solely by name; describe more complex techniques in the Methods section.*
☒	☐	A description of all covariates tested
☒	☐	A description of any assumptions or corrections, such as tests of normality and adjustment for multiple comparisons
☒	☐	A full description of the statistical parameters including central tendency (e.g. means) or other basic estimates (e.g. regression coefficient) AND variation (e.g. standard deviation) or associated estimates of uncertainty (e.g. confidence intervals)
☒	☐	For null hypothesis testing, the test statistic (e.g. F, t, r) with confidence intervals, effect sizes, degrees of freedom and P value noted<br>*Give P values as exact values whenever suitable.*
☒	☐	For Bayesian analysis, information on the choice of priors and Markov chain Monte Carlo settings
☒	☐	For hierarchical and complex designs, identification of the appropriate level for tests and full reporting of outcomes
☒	☐	Estimates of effect sizes (e.g. Cohen's d, Pearson's r), indicating how they were calculated

Our web collection on statistics for biologists contains articles on many of the points above.

Software and code

Policy information about availability of computer code

Data collection	GatorPrime Label-Free Bioanalysis instrumentwas used for binding kinetic studies. Spark multimode microplate reader was used pseudovirus neutralization assay and SpectraMax M4 was used for ELISA. SerialEM software version 3.6 was used for automated cryo-EM image acquisition.
Data analysis	The programs IMGT/V-QUEST (http://www.imgt.org/IMGT_vquest/vquest), IgBLAST (https://www.ncbi.nlm.nih.gov/igblast/), MIXCR (https://mixcr.r eadthedocs.io/en/master/) and VDJtools (https://vdjtools-doc.readthedocs.io/en/master/overlap.html) tools were applied to analyze gene germline, complementarity determining region (CDR) length. Graphs were presented by GraphPad Prism version 9.4; Pymol (https://www.pymol.org/2/) and ChimeraX (https://www.cgl.ucsf.edu/chimerax/) was used to visualize molecular structures. MotionCor2 1.2.1, cryoSPRAC v4.0.1, Relion 3.03, Phenix 1.20.1, Coot 0.9.8, UCSF Chimera 1.16, UCSF Chimera X 1.4, PyMol 2.5.2, ResMap and 3DFSC were used for cryo-EM structural analysis.

For manuscripts utilizing custom algorithms or software that are central to the research but not yet described in published literature, software must be made available to editors and reviewers. We strongly encourage code deposition in a community repository (e.g. GitHub). See the Nature Portfolio guidelines for submitting code & software for further information.

Data

Policy information about availability of data

All manuscripts must include a data availability statement. This statement should provide the following information, where applicable:

- Accession codes, unique identifiers, or web links for publicly available datasets
- A description of any restrictions on data availability
- For clinical datasets or third party data, please ensure that the statement adheres to our policy

The source data for the graphs and charts in the figures is available as Supplementary Data 1. The coordinates and EM map files for the BA.1 spike-1H1 Fab class I complex, BA.1 spike-1H1 Fab class II complex and BA.1 RBD-1H1 Fab local-refined complex have been deposited in the Protein Data Bank (PDB) and the EM Data Bank (EMDB) under accession number PDB-8H00, PDB-8H01 and PDB-8GZZ, and EMDB-34407, EMDB-34408 and EMDB-34406, respectively. The EM map file for the BA.1 spike-1H1 IgG complex has been deposited in the EM Data Bank (EMDB) under accession number EMDB-35328. For materials requests, please reach out to the corresponding authors.

Human research participants

Policy information about studies involving human research participants and Sex and Gender in Research.

Reporting on sex and gender	N/A
Population characteristics	N/A
Recruitment	N/A
Ethics oversight	N/A

Note that full information on the approval of the study protocol must also be provided in the manuscript.

Field-specific reporting

Please select the one below that is the best fit for your research. If you are not sure, read the appropriate sections before making your selection.

☒ Life sciences ☐ Behavioural & social sciences ☐ Ecological, evolutionary & environmental sciences

For a reference copy of the document with all sections, see nature.com/documents/nr-reporting-summary-flat.pdf

Life sciences study design

All studies must disclose on these points even when the disclosure is negative.

Sample size	The number of pseudovirus neutralization assay, ELISA and BLI assay in each group were performed at least 3, which is acceptable in the field. For SPA, 3,177 micrographs of BA.1 spike-1H1 Fab complex and 2,642 micrographs of BA.1 spike-1H1 IgG complex were collected, respectively.
Data exclusions	For SPA, excluded particles were typically obstructed by other particles or contaminants, or in some cases the carbon. For BLI, response data points beyond 800 s were excluded.
Replication	All neutralizing experiments successfully repeated twice with duplicate. The competitive ELISA were performed twice independently. BLI assay for antibody kinetics were performed twice. All attempts at replication were successful.
Randomization	For SPA, particles are randomly oriented, which is confirmed during analysis.
Blinding	N/A

Reporting for specific materials, systems and methods

We require information from authors about some types of materials, experimental systems and methods used in many studies. Here, indicate whether each material, system or method listed is relevant to your study. If you are not sure if a list item applies to your research, read the appropriate section before selecting a response.

Materials & experimental systems

n/a	Involved in the study	
☐	☒	Antibodies
☐	☒	Eukaryotic cell lines
☒	☐	Palaeontology and archaeology
☐	☒	Animals and other organisms
☒	☐	Clinical data
☒	☐	Dual use research of concern

Methods

n/a	Involved in the study	
☒	☐	ChIP-seq
☒	☐	Flow cytometry
☒	☐	MRI-based neuroimaging

Antibodies

Antibodies used	HRP-conjugated Goat F(ab')2 Anti-Rabbit (IgG (Fab')2) secondary antibody (Abclonal, AS014, 1:5000 dilution for ELISA as secondary antibody).
Validation	Primary antibodies reported in this study were described previously in Chen et al. (doi.org/10.1080/22221751.2021.1942227). Target validation was done with multiple binding assays and structural studies using cryo-EM in this article.<br>HRP-conjugated Goat F(ab')2 Anti-Rabbit (IgG (Fab')2) secondary antibody (https://abclonal.com.cn/catalog/AS014).

Eukaryotic cell lines

Policy information about cell lines and Sex and Gender in Research

Cell line source(s)	HEK293-ACE2 (Vzyme, DD1401)<br>FreeStyle 293-F cell (ThermoFisher, R79007)
Authentication	Not authenticated after purchase.
Mycoplasma contamination	We confirm that all cell lines were negative for mycoplasma contamination.
Commonly misidentified lines<br>(See ICLAC register)	No commonly misidentified cell lines were used.

Animals and other research organisms

Policy information about studies involving animals; ARRIVE guidelines recommended for reporting animal research, and Sex and Gender in Research

Laboratory animals	One-month-old female New Zealand Big White Rabbits (Yurogen, Wuhan, China) were utilized for this study.
Wild animals	The study did not involve wild animals.
Reporting on sex	Sex was not considered in study design.
Field-collected samples	The study did not involves sample collected from the field.
Ethics oversight	Animal experiments were approved and operated under the supervision of the Nanjing Drum Tower Hospital Institution Animal Care and Use Committee (IACUC) (protocol No. 2020AE01120).

Note that full information on the approval of the study protocol must also be provided in the manuscript.
